# Lipidomics comparing DCD and DBD liver allografts uncovers lysophospholipids elevated in recipients undergoing early allograft dysfunction

**DOI:** 10.1038/srep17737

**Published:** 2015-12-04

**Authors:** Jin Xu, Ana M. Casas-Ferreira, Yun Ma, Arundhuti Sen, Min Kim, Petroula Proitsi, Maltina Shkodra, Maria Tena, Parthi Srinivasan, Nigel Heaton, Wayel Jassem, Cristina Legido-Quigley

**Affiliations:** 1 Faculty of Life Sciences & Medicine, King’s College London, London, United Kingdom; 2 King’s College Hospital, King’s College London, London, United Kingdom; 3 Department of Analytical Chemistry, Nutrition and Food Science, University of Salamanca, Spain; 4 Institute of Psychiatry, Psychology and Neuroscience, King’s College London, London, United Kingdom

## Abstract

Finding specific biomarkers of liver damage in clinical evaluations could increase the pool of available organs for transplantation. Lipids are key regulators in cell necrosis and hence this study hypothesised that lipid levels could be altered in organs suffering severe ischemia. Matched pre- and post-transplant biopsies from donation after circulatory death (DCD, n = 36, mean warm ischemia time = 21min) and donation after brain death (DBD, n = 76, warm ischemia time = none) were collected. Lipidomic discovery and multivariate analysis (MVA) were applied. Afterwards, univariate analysis and clinical associations were conducted for selected lipids differentiating between these two groups. MVA grouped DCD vs. DBD (*p* = 6.20 × 10^−12^) and 12 phospholipids were selected for intact lipid measurements. Two lysophosphatidylcholines, LysoPC (16:0) and LysoPC (18:0), showed higher levels in DCD at pre-transplantation (*q* < 0.01). Lysophosphatidylcholines were associated with aspartate aminotransferase (AST) 14-day post-transplantation (*q* < 0.05) and were more abundant in recipients undergoing early allograft dysfunction (EAD) (*p* < 0.05). A receiver-o*p*erating characteristics (ROC) curve combining both lipid levels predicted EAD with 82% accuracy. These findings suggest that LysoPC (16:0) and LysoPC (18:0) might have a role in signalling liver tissue damage due to warm ischemia before transplantation.

Liver transplantation is the most viable solution to a range of acute and chronic end-stage liver diseases^1^. The global prevalence of liver disorders such as cirrhosis^2^, hepatitis B and C^3^,^4^ and non-alcoholic fatty liver disease^5^ have resulted in a marked increase in the demand for transplantation. However, widespread shortfalls in donor organ availability mean that the demand for transplantation greatly exceeds its actual occurrence^1^. This crippling donor shortage has led to the increase use of organs from ‘marginal’ donors^6^,^7^, including those obtained from donation after circulatory death (DCD). The use of DCD livers remains limited as organs are exposed to a significant period of warm ischemia prior to retrieval and have poorer patient outcomes^8^,^9^,^10^. It is estimated that up to a fifth of donation after brain death (DBD) organs do not meet the strict clinical criteria for transplantation, and are thus discarded^11^, with higher losses reported for DCD grafts^12^. A primary goal of the pre-transplantation donor evaluation is determining whether the donor liver is more susceptible to graft dysfunction following transplantation^13^,^14^. While the assessment is an important patient safeguard, it may also result in otherwise transplantable organs being discarded. Increasing the pool of available and transplantable livers by identifying specific pre-transplantation markers of liver damage is thus a high priority^15^,^16^.

Elevated liver-enzyme levels are widely accepted as the standard for liver injury, however these tests lack specificity as they can be affected by medication and other syndromes^17^. Biomarker discovery in the context of liver pathophysiology has been predominantly genomic and transcriptomic-based^18^. In order to find metabolite markers in liver tissue, targeted and metabolite phenotyping strategies have been applied to find markers relevant to liver transplantation^19^,^20^, findings highlighted lipid associations to early allograft dysfunction (EAD) and recipient clinical outcomes^14^,^21^,^22^,^23^.

Here, we investigate lipid fingerprints at both pre- and post-transplantation, using ultra-performance liquid chromatography-mass spectrometry (UPLC-MS) in hepatic tissue in two distinct donor types: *viz.* DBD and DCD, the latter undergoing warm ischemia events^24^ . The main lipid differences between these two donor types were determined by an initial lipidomics screen (112 biopsies), which highlighted 12 targeted phospholipids that were reanalysed in targeted mode and univariate comparisons. After this, associations to clinical outcomes were investigated. The study workflow is illustrated in Fig. 1.

## Results

### Clinical outcomes

There were no significant differences between DBD and DCD groups in donors’ ages, EAD/ immediate graft function (IGF) distribution, liver enzymes, hepatic steatosis or serum bilirubin levels. Differences were observed in the recipients’ ages (*p* < 0.01) between these two groups (Table 1).

### Lipidomics multivariate analysis and selected phospholipids

**Figure 2:**
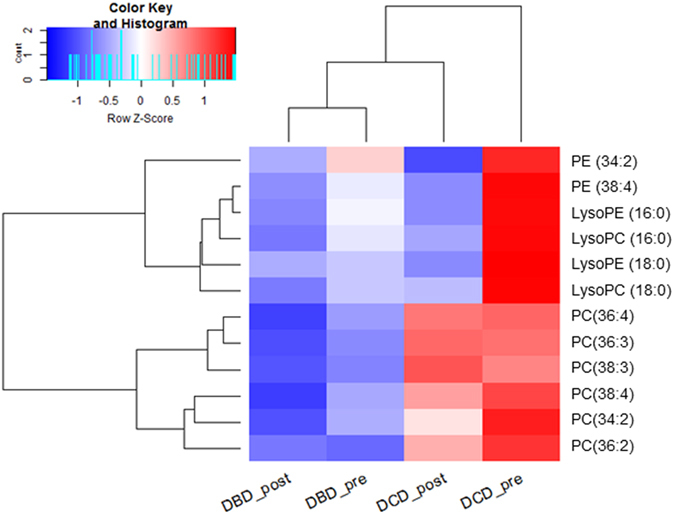
Heat-map showing distinct lipid profiles of DBD and DCD tissue (n = 112). Values are median amounts per donor group at pre and post-transplantation stages. A clustering analysis (dendrogram) shows which lipids differ most; red depicts higher levels and blue means lower levels. DCD, donation after circulatory death; DBD, donation after brain death; PE, phosphatidylethanolamine; PC, phosphatidylcholine; LysoPC, lysophosphatidylcholine; LysoPE, lysophosphatidylethanolamine.

To discover relevant lipid features, an orthogonal projections to latent structures-discriminant analysis (OPLS-DA) model (Supplementary Figure S1) among DBD and DCD grafts was built with n = 112. The model’s figures of merit were R^2^X = 0.659, R^2^Y = 0.941 and Q^2^ = 0.58. R^2^X explains a feature percentage (65.9%) which is explained by this model, R^2^Y indicates that 94.1% of the group variance is interpreted, and Q^2^ shows the prediction ability of the model with 58%. 7-fold cross validation (CV) (CV *p* value = 6.20 × 10^−12^) suggested its reliability. From the model, 12 features differentiated among 2 groups with p[1]>0.1, p(corr)>0.4 & p[1]<−0.05, p(corr)<−0.18 were selected (Supplementary Table S1). These selected 12 features consisted of 2 lysophosphatidylethanolamines (LysoPEs), 2 lysophosphatidylcholines (LysoPCs), 6 phosphatidylcholines (PCs) and 2 phosphatidyl-ethanolamines (PEs). Donor age, steatosis status, functional warm ischemia time (WIT) and cold ischemia time (CIT) showed low p[1] and p(corr) values and were not chosen as important variables. A heat map was computed to visualise trends for the 12 selected lipids (Fig. 2).

### Targeted analysis of phospholipids per donor type during transplantation

From the heat map, no obvious differences were observed in the DBD group from pre- to post- for all 12 lipids except for PE (34:2). All lipids were more abundant in DCD at pre-transplantation stage compared with DBD. All 6 PCs levels remained constant from pre to post in the DCD group while 2 PEs, 2 LysoPEs and 2 LysoPCs showed lower concentration at post- transplant stage.

**Figure 3:**
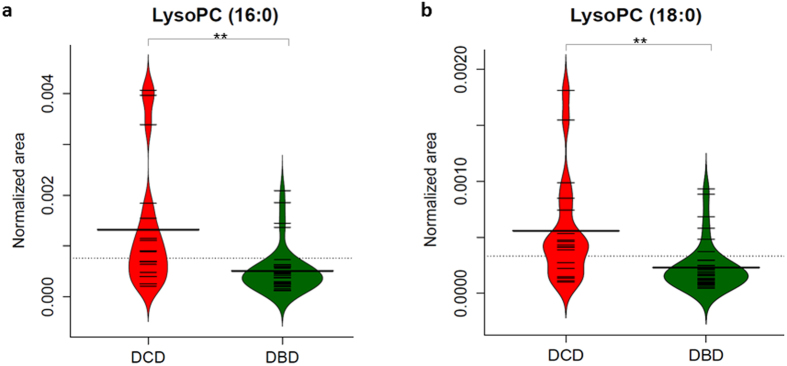
Bean-plots show levels for two lysophosphocholines (Mann-Whitney 2-sided, ** is *q* < 0.01, q value is p value adjusted by Benjamini and Hochberg FDR correction) among DCD and DBD at the pre-transplantation stage. (a) LysoPC (16:0); (b) LysoPC (18:0). DCD, donation after circulatory death; DBD, donation after brain death; LysoPC, lysophosphatidylcholine.

12 lipids were reanalysed from the semi-quantified data for univariate analysis. The result of Mann-Whitney test with multiple comparison correction revealed that 2 lysophosphatidylcholines showed significant differences at pre transplantation stage (*q* = 0.002 and *q* = 0.003 respectively) between the two donor groups. Figure 3 shows the amounts for LysoPC (16:0) and LysoPC (18:0), Figs. 3a,b respectively show elevated levels in DCD when comparing to DBD for both LysoPCs (*q* < 0.01).

### Correlation of selected lysophospholipids to clinical data

**Figure 4:**
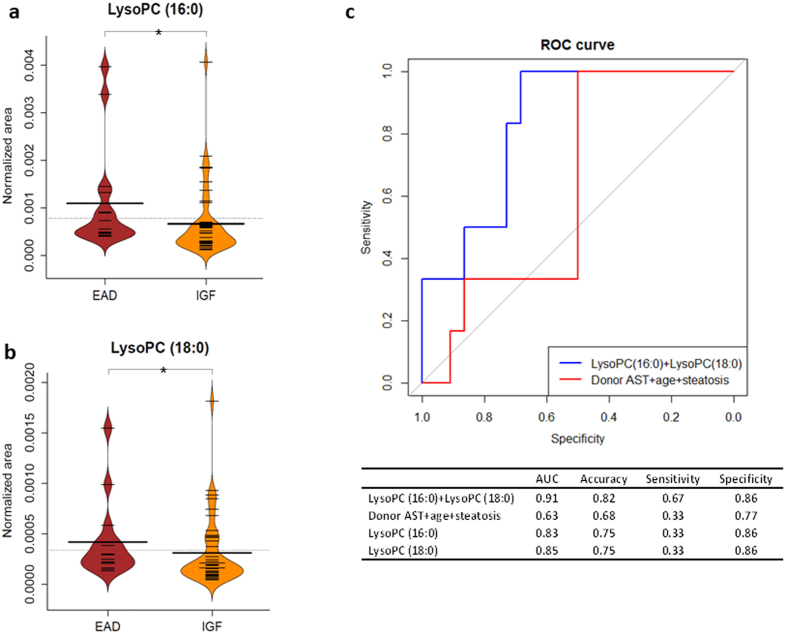
Two LysoPCs amounts, (**a**) LysoPC (16:0) and (**b**) LysoPC (18:0), showing significant differences between EAD (n = 15) and IGF (n = 41) groups (Mann-Whitney 2-sided, is *p < 0.05); (**c**) ROC curve prediction of EAD based on two LysoPCs and three donor clinical parameters. LysoPC, lysophosphatidylcholine; EAD, early allograft dysfunction; IGF, immediate graft function; ROC, receiver operating characteristic.

The distribution of 2 lysophosphatidylcholines in EAD (n = 15) and IGF (n = 41) groups was investigated. The Mann-Whitney test illustrated that the amount of LysoPC (16:0) and LysoPC (18:0) at pre-transplant were significantly higher in the EAD group (*p* = 0.013 and *p* = 0.03 respectively) (Fig. 4a,b). The prediction ability of LysoPCs (LysoPC (16:0) & LysoPC (18:0)) and clinical parameters (donor AST, donor age and steatosis status) was evaluated by receiver-operating characteristics (ROC) curve. The area under curve (AUC) for LysoPCs group was 0.91 (accuracy = 0.82, sensitivity = 0.67, specificity = 0.86). For comparison, the AUC for three pre-transplant clinical parameters was 0.63 (accuracy = 0.68, sensitivity = 0.33, specificity = 0.77) (Fig. 4c).

The associations of the 2 selected pre-transplant lysophosphatidylcholines levels on biomedical parameters including AST, bilirubin, and creatinine levels within 14-day post-transplant were examined by mixed-effects maximum likelihood regression followed by Benjamini and Hochberg correction. Overall 6 models were built with the combination of one biomedical parameter and one lipid in each model and adjusted for age and gender. Fitting was deemed adequate for all 6 models (p < 0.0001 from chi-square test). After multiple comparison correction, significant associations were observed between longitudinal changes in AST concentration (slope) and LysoPC (16:0) levels (LysoPC (16:0) × day interaction (q < 0.05)), and between longitudinal changes in AST concentration (slope) and LysoPC (18:0) levels (LysoPC (18:0) × day interaction (q < 0.05)). For instance, with every standard deviation (SD) increase in LysoPC (16:0) × day interaction, recipients’ AST levels, on average, decreased 0.017 IU/L (with 95% confidence interval subscripts: LysoPC (16:0) × day interaction: _-0.032_−0.017_−0.003_ p = 0.006). The model parameters are listed in Table 2 and the correlation between LysoPCs concentrations and the AST levels are present in Supplementary Figure S2.

## Discussion

The urgent need for specific molecular markers of hepatic tissue quality has given rise to small molecule phenotyping studies investigating a range of liver pathologies^14^,^25^. The study reported here is the first study to distinguish lipid profiles between two different liver-donor types, with correlations to clinical outcomes related with liver graft dysfunction and clinical follow-up. Matched liver tissue biopsies were obtained at both pre- and post-transplantation stages from two types of donors, *viz.* DBD and DCD. DBD livers may suffer inflammatory changes in relation to brain death and ITU management, and undergo a significant period of cold preservation following retrieval. DCD donors have no diagnosis of brain death; however, livers from DCD undergo an additional period of warm ischemia prior to retrieval. This ischemia period has been previously associated with increased rates of graft failure and with both short and long-term complications following transplantation^10^. A substantial number of DCD organs are thus discarded because of the lack of precise assays to evaluate transplant outcomes.

Inflammatory responses in donor liver biopsies are donor-type specific, DBD tissue showed high levels of pro-inflammatory changes at the pre-transplantation stage. This was attributed to inflammatory events associated with brain death in the donors^26^. Following reperfusion, DBD tissue showed high levels of neutrophil infiltration and deposition of activated platelets. On the other hand, DCD allografts demonstrated lower inflammatory response but higher cell death rates that correlated with the length of warm ischemia^26^,^27^. Since increasing cell death was observed in DCD, we hypothesised that lipid cell death mediators could be affected during transplant^28^.

By focusing on donor-type using lipidomics for discovery, we identified 12 lipids differentiating among DBD and DCD. Trends were illustrated in the heat-map (Fig. 2) for the lipid median values in pre- and post- transplantation across the two donor types. This panel of lipids did not change from pre- to post-transplant in the same donor type, implying that lipid changes observed at pre-transplantation are likely to be related to ischemia damage rather than reperfusion injury.

PCs were more abundant in DCD at both transplant stages. Phosphatidylcholines have been associated with inflammation^29^. However, contradictory data shows that these lipids could have a protective function as studies have linked them to regeneration processes in the liver^30^,^31^,^32^.

Twelve lipids were measured again and univariate analysis was applied. Lipids with statistical significance when comparing DBD and DCD in both pre- and post-transplant biopsies were LysoPC (16:0) (*q* < 0.01) and LysoPC (18:0) (*q* < 0.01) showing higher values in DCD (Fig. 3).

**Figure 5:**
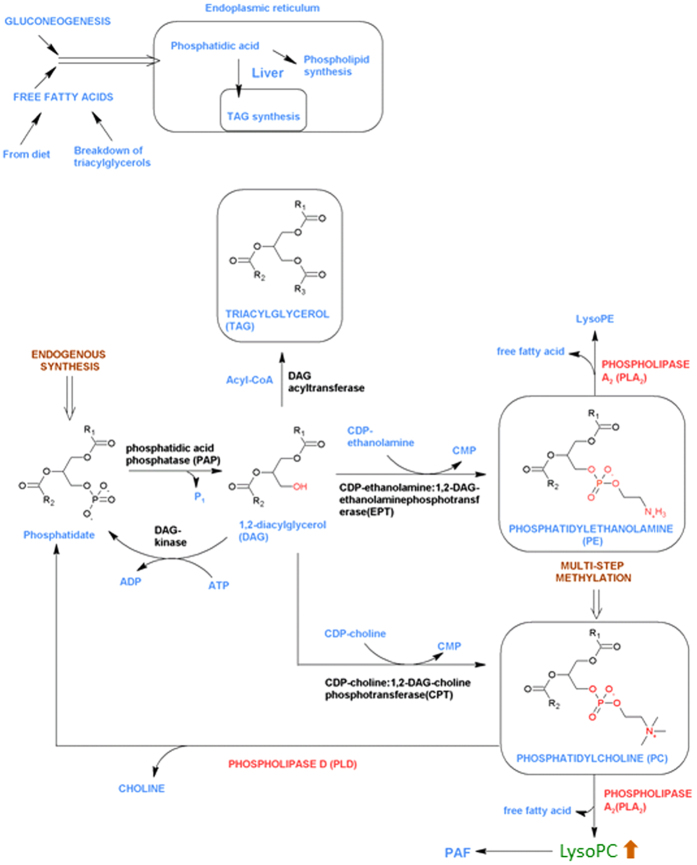
Lipid metabolism in the healthy liver, showing endogenous metabolism of triacylglycerol (TAG) and phospholipids. Phosphatidic acid (PA) is generated *in vivo* by catabolic modification of glycerol-3-phosphate. Diacyglycerol (DAG) is a key metabolic intermediate and intracellular signalling molecule which can be converted to TAGs by the action of DAG acyltransferases to glycerophospholipids including phosphatidylcholines (PC), phosphatidylethanolamines (PE) and ultimately to lysophosphatidylcholines (LysoPC), lysophosphatidylethanolamines (LysoPE) by the action of phospholipase A_2_. LysoPCs showed higher amounts in donation after circulatory death (DCD) group in this study.

LysoPCs are lysophospholipids generated from PCs (Fig. 5) via the action of phospholipase A_2_, and like other lysophospholipids they are likely to be activators or inhibitors of G-protein coupled receptors (GPCRs)^33^. Phospholipase A_2_ synthesises lysophospholipids including both lysoPCs and lysoPEs from PEs and PCs by producing free fatty acids.

In DCD donors, LysoPCs were found to be increased. LysoPCs are known to be precursors of the platelet-activating factor (PAF), a potent phospholipid inflammatory, which has been previously associated with both hepatic ischemia and reperfusion injury (IRI)^34^,^35^,^36^. Hence, DCD donors might be prone to the PAF mediated inflammatory pathway before transplantation (pathway illustrated in Fig. 5).

The *in-vivo* function of LysoPCs and in particular their role in intracellular signalling is mostly unknown. LysoPCs have previously been associated with inflammatory liver disease^37^,^38^. Interestingly Cortes *et al.*^14^ found in a study of liver transplantation with DBD grafts that LysoPC (16:0) and LysoPC (18:0) were higher in grafts that presented EAD *p* < 0.05, supporting the hypothesis that these lysophospholipids affect transplant outcomes.

To understand the relevance of the two lipids to transplantation we investigated EAD. EAD is a clinical term which can reflect donor, recipient and transplant characteristics, and it can be utilized as a transplant benchmark^39^. The incidence of EAD in our study was 26%, this was in the upper range of known values for incidence ranging from 14% to 27%^40^. LysoPC (16:0) and LysoPC (18:0) showed (*p* < 0.05) significant difference when their contents between EAD and IGF groups were compared. The ROC curve of these two LysoPCs versus three clinical parameters known to be risk factors of EAD indicated that LysoPC (16:0) and LysoPC (18:0) were better at prediction of EAD, in particular accuracy increased from 68% for AST, age and steatosis to 82% for LysoPCs. A limitation of this comparison is that it was applied to compare donors only at pre-transplantation; however it is known that EAD is affected by multiple factors including the recipients and the surgery procedures^41^.

The regression between AST 14-day concentrations and two LysoPCs indicated that LysoPCs levels at pre-transplant were associated to AST concentration at post-transplant. The model indicated LysoPCs’ association with higher AST immediately after transplant. Since AST is considered an indicator of liver injury, the association between high AST post-transplantation and LysoPCs pre-transplantation warrants further investigation of LysoPC (16:0) and LysoPC (18:0) as markers of liver damage.

In conclusion, the analysis of phospholipids in the context of liver transplantation has identified two lipids differentiated in DBD and DCD livers. LysoPC (16:0) and LysoPC (18:0) could have a role as intermediates in signalling tissue damage due to warm ischemia. This study is relevant in identifying pre-transplant biomarkers for tissue quality and in designing appropriate therapeutic strategies in order to minimize damage related to ischemia injury.

## Patients and Methods

### Patients and biopsy collection

This study received prior approval from the ethics committee at King’s College Hospital, and informed consent was obtained from all subjects. The methods were carried out in accordance with the approved guidelines. Overall 112 Tru-Cut tissue biopsies were obtained from liver allografts pre- and post-transplantation. The first (pre-transplant) biopsy was taken at the end of cold preservation, prior to implantation, and the second (post-transplant) biopsy was obtained approximately 1 hour after graft reperfusion. A separate biopsy was obtained for histopathological evaluation of donor steatosis. Biopsies were immediately snap-frozen in liquid nitrogen and stored at –80°C until extraction for LC-MS analysis. In all procedures, liver allografts were flash-cooled and perfused with University of Wisconsin preservation fluids until the time of transplantation.

Power calculations were performed for DBD (n = 38) and DCD (n = 18) participants using “Gpower3.1” (http://www.gpower.hhu.de/). Assuming a two-sided Type I error of 0.05 and standard normal distributions for lipid molecules, this study has >80% power to detect differences between two groups based on our previous work^21^.

### Donors

The study included two types of adult donors: DBD (n = 38) and DCD (n = 18). A wide spectrum of donor clinical data was collected for comparison among groups and for correlation with lipid levels. In the DBD group, 18 of the livers had mild steatosis (up to 30% fat on biopsies), 6 had moderate steatosis (30–60% fat) and the remaining DBD grafts had none. In the DCD group, 2 allografts were mildly steatotic, 6 allografts were moderately steatotic and the remainder were normal. In the DCD group, WIT was calculated from the time when systolic blood pressure was below 50 mmHg to the time of cold perfusion. Total WIT is the sum of Functional WIT, Hepatectomy time and Bench perfusion. The relevant donor data are included in Table 1.

### IGF

#### Recipients

All recipients were patients with stable chronic liver disease who did not require hospitalization prior to transplantation. Indications of liver transplantation in the study include alcoholic liver disease (ALD), primary sclerosing cholangitis (PSC), hepatitis C virus (HCV), hepatocellular carcinoma (HCC), biliary atresia (BA) and others. After transplantation, all patients received immunosuppressive therapy with tacrolimus and prednisolone. Recipients’ 14-day period of international normalized ratio (INR), AST, albumin, gamma-glutamyl transferase (GGT), bilirubin, alkaline phosphatase (ALP) and creatinine were recorded. Graft performance was assessed based on AST, INR and bilirubin levels after transplantation^42^. According to graft performance, recipients were classified into two groups: showing EAD (n = 15) and IGF (n = 41). The relevant recipient details are listed in Table 1.

#### Lipidomics

Sample preparation for all 112 biopsies and lipidomic analysis followed our previously published method^43^. Lipidomics was performed using Waters ACQUITY ultra-performance liquid chromatography-quadrupole time of flight (UPLC-QToF) in both positive and negative ionisation mode. All data was processed within XCMS in R and exported into SIMCA version 13 (MKS Umetrics AB, Sweden) for multivariate analysis. Multivariate analysis included pre- and post-transplant matched samples n = 112 (DBD n = 76, DCD n = 36). Donor age, steatosis status, WIT and CIT were included as X-variables for multivariate analysis. OPLS-DA was then performed to select features based on covariance p[1] and correlation p(corr) value (p[1]>0.1, p(corr)>0.4 & p[1]<-0.05, p(corr)<-0.18).

#### Intact Lipid Analysis

Selected lipids were measured in the LC-MS data using Waters MassLynx software (Waters Corporation, Milford, MA) and peak areas were normalized to total ion count ratios. The identification was performed by structure and fragmentation patterns in the MS^2^ data^44^,^45^,^46^. Supplementary Table S1 contains the relevant analytical data for the lipid panel analysis (list of mass-to-charge ratio or m/z values, the observed ions and the instrumental variation of the measurements in QC samples).

Median values were used to plot the heat-map using an open source ‘R’ with ‘gplots’ package^47^, bean-plots were drawn in ‘beanplot’ package^48^. Levels in each group at pre- and post-transplantation stage as well as between DCD and DBD groups were examined with univariate non-parametric Mann-Whitney test (2-sided) with Benjamini and Hochberg correction to control for false discovery^49^. All *p* values were represented as *q* values after correction. Lipids with *q* < 0.01 were selected for clinical correlation analysis.

#### Clinical correlations

The selected lipid levels in both EAD and IGF groups were investigated with Mann-Whitney test (2-sided) to examine any lipid distribution differences, calculations were conducted in SPSS 22 (IBM: Armonk, United States). A prediction model in regard to EAD was built using ROC curve for two combined LysoPCs (LysoPC (16:0) & LysoPC (18:0)) and three combined clinical data (donor AST, donor age and steatosis status) in ‘R’ with ‘pROC’ package. The prediction ability of these two curves was assessed by area under curve, accuracy, sensitivity and specificity.

Mixed linear mixed effects models were used to investigate the longitudinal associations between recipients’ 14-day AST, bilirubin and creatinine concentrations and each lipid pre-transplant levels. Data were scaled to obtain standard deviation of 1. The average baseline AST, bilirubin and creatinine concentrations and the average change in their concentrations over 14 days (follow-up time) were calculated for all subjects per visit (day) as a group (fixed effects) and subject-specific intercept and slope terms, which reflected deviation from the group average (mixed linear effects) were calculated. An interaction term between visit (day) and lipid pre-transplant levels was used to investigate whether the recipients’ longitudinal AST, bilirubin and creatinine concentrations (slope) was associated with lipid pre-transplant levels. All calculations included adjustment for age and gender for the 56 donors. All obtained p values were corrected for multiple comparisons and results given as q values. Linear mixed effect models were performed in Stata/SE13 (StataCorp: Texas, United States).

## Additional Information

**How to cite this article**: Xu, J. *et al.* Lipidomics comparing DCD and DBD liver allografts uncovers lysophospholipids elevated in recipients undergoing early allograft dysfunction. *Sci. Rep.* **5**, 17737; doi: 10.1038/srep17737 (2015).

## Supplementary Material

Supplementary Information

## Figures and Tables

**Figure 1:**
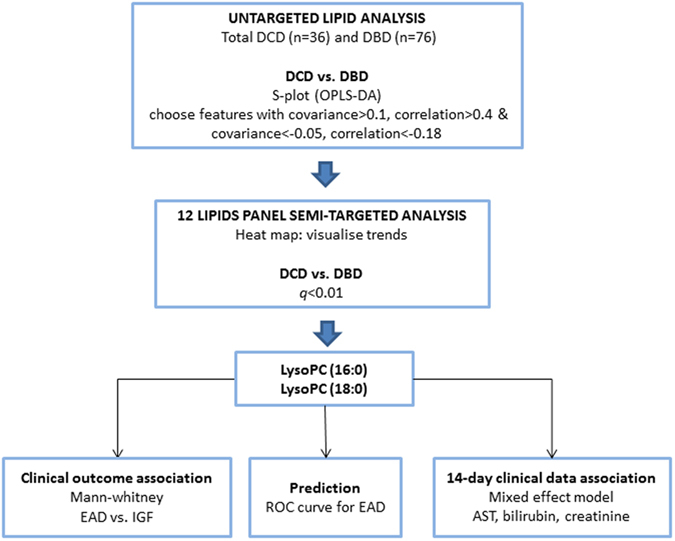
Study flowchart illustrates the overall design from untargeted analysis to semi-targeted analysis and association of potential biomarkers to clinical outcomes. DCD, donation after circulatory death; DBD, donation after brain death; OPLS-DA, orthogonal projections to latent structures-discriminant analysis; LysoPC, lysophosphatidylcholine; CIT, cold ischemia time; WIT, warm ischemia time, EAD, early allograft dysfunction; IGF, Immediate Graft Function; AST, aspartate aminotransferase.

**Table 1:**
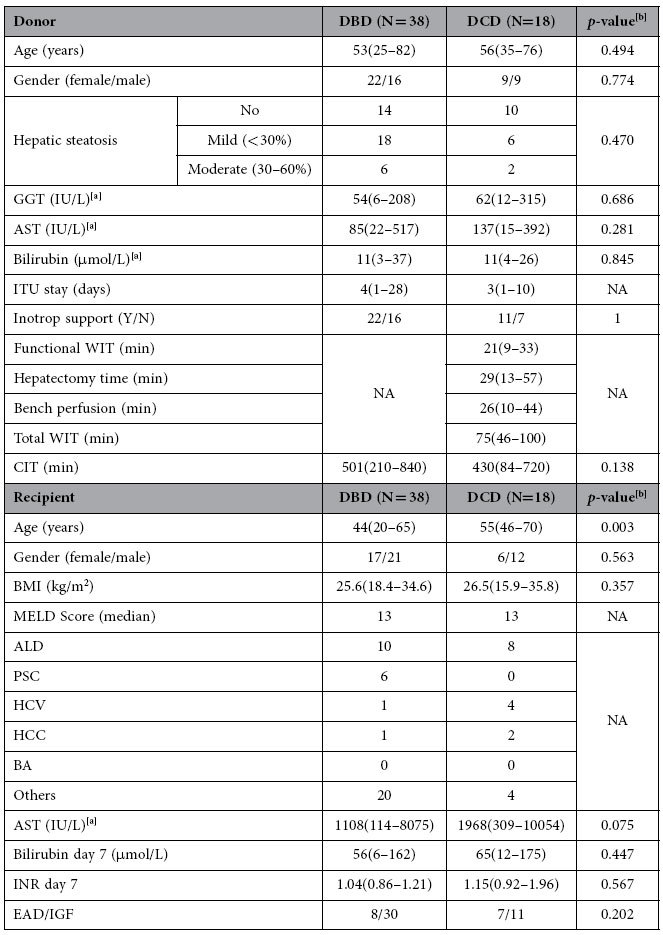
Summary of clinical data for liver donors and recipients.

DBD, donation after brain death; DCD, donation after circulatory death; GGT, gamma-glutamyl transferase; AST, aspartate aminotransferase; ITU, intensive therapy unit; WIT, warm ischemia time; CIT, cold ischemia time; BMI, body mass index; MELD, model for end-stage liver disease; ALD, alcoholic liver disease; PSC, primary sclerosing cholangitis; HCV, hepatitis C virus; HCC, hepatocellular carcinoma; BA, biliary atresia; EAD, early allograft dysfunction; IGF, immediate graft function.

Continuous values are expressed as means (minimum-maximum); NA, not applicable.

Total WIT is the sum of Functional WIT, Hepatectomy time and Bench perfusion.

^a^Tested on the day of operation.

^b^Mann Whitney test (2-sided) or Fisher exact test (2-sided).

**Table 2:** Mixed-effect models summarizing the baseline and longitudinal associations between LysoPC levels and AST concentration. LysoPC(16:0) and LysoPC(18:0) indicated baseline associations (intercept) and the interaction with day indicates longitudinal associations (slope).

AST		Coefficient	95% confidence interval	*p*-value
Model 1	LysoPC (16:0)	0.122	−0.068, 0.313	0.21
	day	−0.0003	−0.016, 0.015	0.97
	LysoPC(16:0) × day	−0.017	−0.032, −0.003	**0.006**
Model 2	LysoPC (18:0)	0.117	−0.073, −0.308	0.229
	day	−0.0003	−0.016, 0.015	0.965
	LysoPC(18:0) × day	−0.02	−0.035, −0.005	**0.007**

LysoPC, Lysophosphatidylcholine; AST, aspartate aminotransferase.
