# Supplementary material for: Lipidomics comparing DCD and DBD liver allografts uncovers lysophospholipids elevated in recipients undergoing early allograft dysfunction
Source: Sci Rep. 2015 Dec 4;5:17737. doi: 10.1038/srep17737 (PMC4669413; doi:10.1038/srep17737)
Supplement: Supplementary Information [file srep17737-s1.pdf]

Supplementary material for:

Lipidomics comparing DCD and DBD liver allografts uncovers lysophospholipids elevated in recipients undergoing early allograft dysfunction

Jin Xu[+], Ana M. Casas-Ferreira[+], Yun Ma, Arundhuti Sen, Min Kim, Petroula Proitsi, Maltina Shkodra, Maria Tena, Parthi Srinivasan, Nigel Heaton, Wayel Jassem*****, Cristina Legido-Quigley*****

[+] These authors contributed equally to this work.

*****Correspondence and requests for materials should be addressed to:

Cristina Legido-Quigley: Faculty of Life Sciences & Medicine, King's College London, London SE1 9NH, United Kingdom. Tel.: +44 020 78484722. E-mail: cristina.legido_quigley@kcl.ac.uk

Wayel Jassem: King's College Hospital, King's College London, London SE5 9RS, United Kingdom. Tel.: +44 020 32999000 ex31144. E-mail: wayel.jassem@kcl.ac.uk

Preparation of extracts

Between 10 and 30 mg of tissue were obtained and transferred to a pre-weight Eppendorf tube containing a steel bead. Then, 30 µL (per 10 mg of tissue) of a methanol: water mixture (4:1, containing the negative-mode internal standard heptadecanoic acid 10 µg/mL) was added and the samples were homogenized for 5 min at 25 Hz (10 cycles of 0.5 min) in a TissueLyser (Qiagen, MD, US). Following this, 70 µl of the homogenate was added to an amber glass HPLC vial containing a 300 µl glass insert (Chromacol, UK). To this 200 µl of Methyl tert-butyl ether (MTBE) containing 10 µg/mL of positive-mode internal standard (tripentadecanoin) was added, and the samples were mixed via vortexing at room temperature for 60 min. Then, 30 µl of high purity water was added and samples were centrifuged at 2500g for 20 min at 4 ºC. Another 35 µl of the homogenate from each sample was taken to form the pooled QC samples. Same procedures were followed for QCs extraction. The upper lipid-containing MTBE phase was then injected onto the LC-MS system directly from the vial. Two different ionization modes including positive and negative were performed for each sample. QC sample was running in between every 8 samples.

Chromatographic and mass spectrometry conditions

Chromatographic separation for lipidomics (phase one and two) was achieved using an Agilent Poroshell 120 EC-C8 column (150mm × 2.1mm, 2.7 µm), maintained at 55 °C. A gradient was employed consisting of 10 mM ammonium formate in water (A) and 10mM ammonium formate in methanol (B). The solvent was delivered at a flow rate of 0.5 mL/min. The gradient consisted of 0 min (75% B), 23 min (96% B), 36 min (96% B), 36.5 min (100% B), and 41.5 min (100% B). The column was re-equilibrated at 75% B for 9 minutes prior to each injection. The mass spectrometer (Xevo-QTOF) was operated in positive and negative ion mode. For positive mode, a capillary voltage of 3.2 kV and a cone voltage of 45 V were

used. The desolvation gas flow was 400 L/hour and the source temperature was 120°C. All analyses were acquired using the lock spray to ensure accuracy and reproducibility; leucine enkephalin was used as lock mass (m/z 556.2771 and 278.1141) at a concentration of 200 ng/mL and a flow rate of 10 μL/min. Data were collected in the centroid mode over the mass range m/z 100–1000 with an acquisition time of 0.1 seconds a scan. For negative mode, a capillary voltage of -2.6 kV and a cone voltage of 45 V were used. Desolvation gas flow and source temperature were fixed at 800 L/h and 350 ºC, respectively. A reference solution (leucine enkephalin) was infused at regular intervals, and use to update accurate mass data values.

Supplementary Table S1 Identification of markers based on molecular weight, retention time and collision induced dissociation fragmentation of 12 lipids.

m/z	Retention time (min)	Identified as	Observed ion	QC RSD (%)
452.28	3.46	LysoPE (16:0)	$[M-H]^-$	9.91
480.31	5.24	LysoPE (18:0)	$[M-H]^-$	7.86
496.33	3.42	LysoPC (16:0)	$[M+H]^+$	10.9
524.37	5.16	LysoPC (18:0)	$[M+H]^+$	20.3
714.51	16.81	PE (34:2)	$[M-H]^-$	4.61
750.55	19.32	PE (38:4)	$[M-H]^-$	4.51
758.56	16.73	PC (34:2)	$[M+H]^+$	19.5
782.57	16.80	PC (36:4)	$[M+H]^+$	8.92
784.59	17.38	PC (36:3)	$[M+H]^+$	7.66
786.61	18.41	PC (36:2)	$[M+H]^+$	10.6
810.60	18.46	PC (38:4)	$[M+H]^+$	1.77
812.62	19.01	PC (38:3)	$[M+H]^+$	11.4

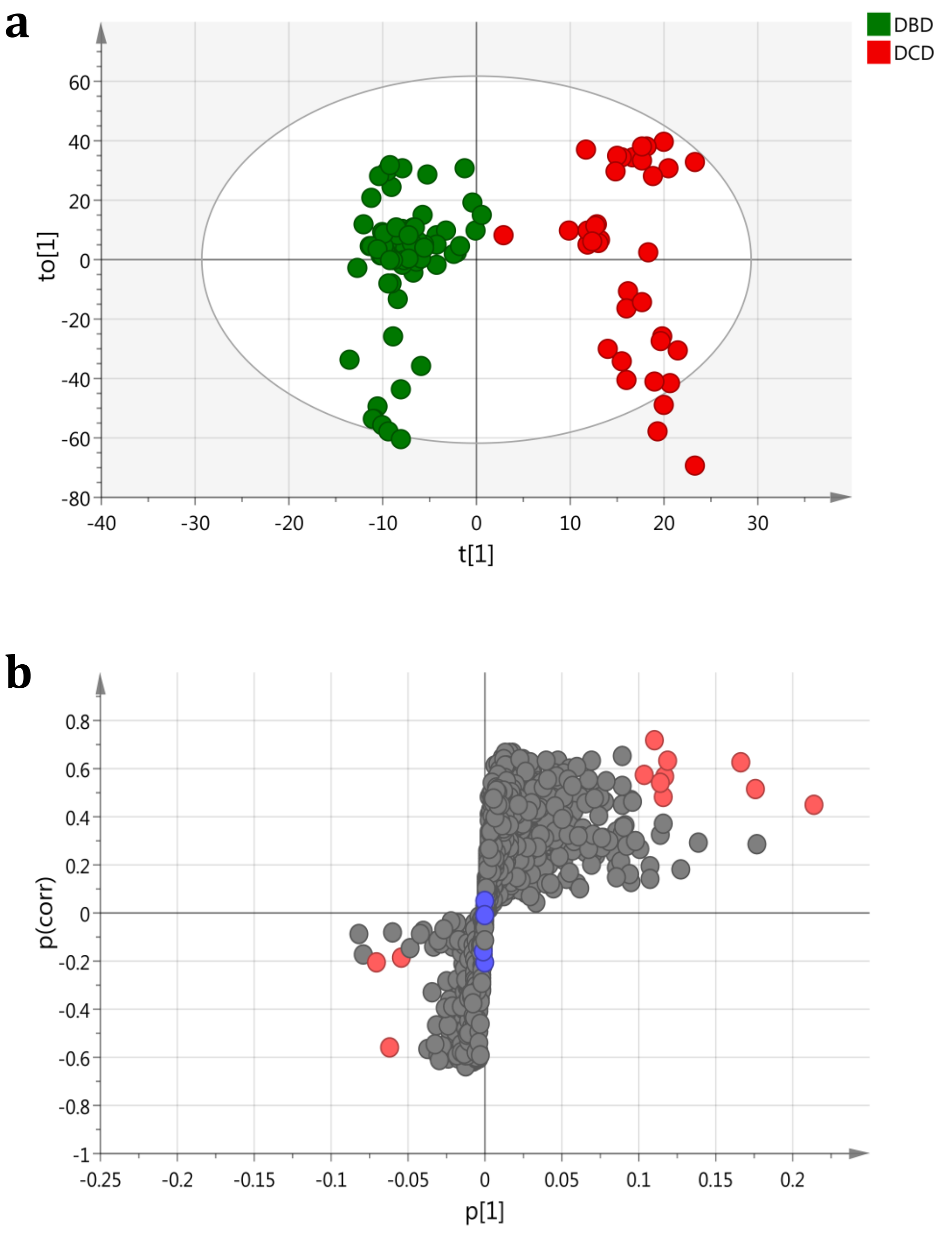

Supplementary Figure S1 OPLS-DA model from screen of lipidomics with n=112 (DBD=76, DCD=36) biopsies. a) Score plot of the model, $R^2X=0.659$, $R^2Y=0.941$, $Q^2=0.58$, cross validation *p* value=6.20×10^{-12}; b) S-plot of the model, features with p[1]>0.1, p(corr)>0.4 & p[1]<-0.05, p(corr)<-0.18 were selected (red circles), variables including donor age, steatosis status, WIT and CIT were clustered in the middle (blue circles).

a

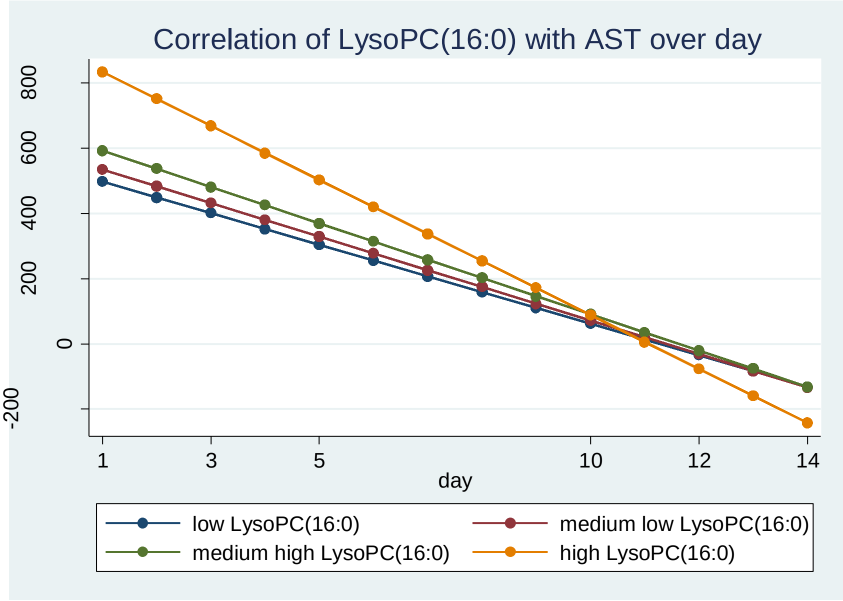

b

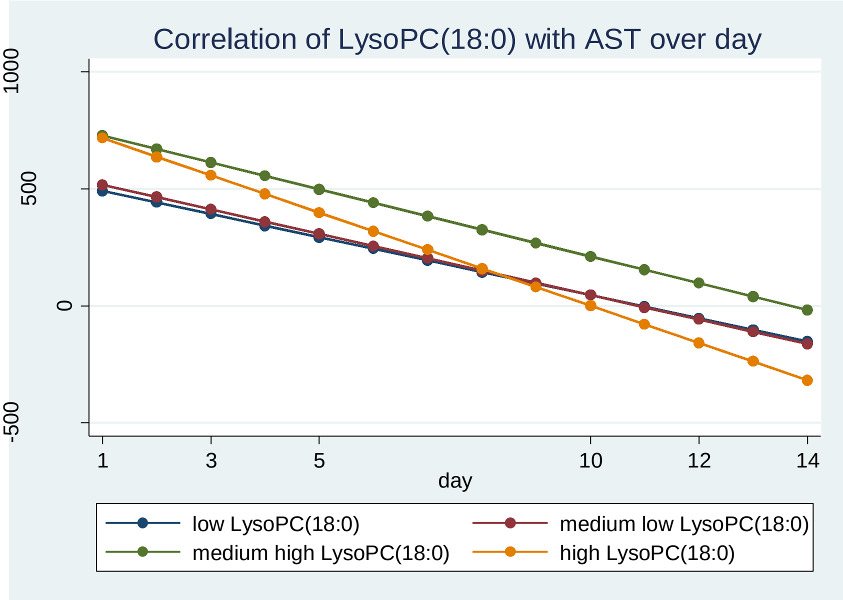

Supplementary Figure S2 Correlation of two pre-transplant lysophospholipids level and post-transplant AST, bilirubin and creatinine during 14-day period in mixed-effects model. a) Correlation of LysoPC (16:0) and AST over 14-day; b) Correlation of LysoPC (18:0) and AST over 14-day.
